# Maternal obesity and labour complications following induction of labour in prolonged pregnancy

**DOI:** 10.1111/j.1471-0528.2010.02889.x

**Published:** 2011-01-26

**Authors:** S Arrowsmith, S Wray, S Quenby

**Affiliations:** a Department of Cellular and Molecular Physiology, Institute of Translational Medicine, University of LiverpoolLiverpool, UK; b Clinical Sciences Research Institute, University of WarwickWarwick, UK

**Keywords:** Body mass index, induction of labour, labour complications, obesity, prolonged pregnancy

## Abstract

**Objective:**

To investigate the effect of maternal obesity on mode of delivery following induction of labour (IOL) for prolonged pregnancy and subsequent intrapartum and neonatal complications.

**Design:**

Retrospective (historical) cohort study.

**Setting:**

Liverpool Women's Hospital NHS Foundation Trust, UK.

**Population:**

A total of 29 224 women with singleton pregnancies between 2004 and 2008 of whom 3076 had a prolonged pregnancy (defined as ≥290 days or 41^+3^ weeks of gestation) and received IOL.

**Methods:**

Kruskal–Wallis test, chi-square test and multivariable logistic regression.

**Main outcome measures:**

Mode of delivery and risk of delivery and neonatal complications in obese verses non-obese women following IOL.

**Results:**

Obese women had a significantly higher rate of IOL ending in caesarean section compared with women of normal weight following IOL (38.7% versus 23.8% primiparous; 9.9% versus 7.9% multiparous women, respectively); however, length of labour, incidence of postpartum haemorrhage and third-degree tear, rate of low cord blood pH, low Apgar scores and shoulder dystocia were similar in all body mass index categories. Complications included a higher incidence of fetal macrosomia and second-degree, but not third-degree, tear in primiparous women.

**Conclusions:**

Higher maternal body mass index at booking is associated with an increased risk of prolonged pregnancy and increased rate of IOL. Despite this, more than 60% of obese primiparous and 90% of multiparous women with prolonged pregnancies who were induced achieved vaginal delivery and labour complications in the obese women with prolonged pregnancies were largely comparable to those of normal weight women with prolonged pregnancies. Our data suggest that IOL for prolonged pregnancy in obese women is a reasonable and safe management option.

## Introduction

The prevalence of obesity has risen such that it is now a worldwide epidemic.^1^ As obesity increases, so does the number of women of reproductive age who are overweight and obese. This is having deleterious effects on female reproduction in general and a major impact on maternity services.^2^–^4^ In the UK, it is now estimated that one in five women at antenatal booking are obese.^5^ Many studies have demonstrated that obesity in pregnancy is associated with a wide spectrum of adverse pregnancy outcomes including increased caesarean section rates, postpartum haemorrhage, higher risks of maternal hypertension and gestational diabetes and fetal death.^2^,^3^,^6^–^9^ Obesity in pregnancy has also been shown to be associated with longer gestation^10^ and significantly increased risk of post-term delivery,^11^–^14^ which contributes to the greater need for induction of labour (IOL) for prolonged pregnancy.^15^–^17^ As gestation progresses beyond term, perinatal morbidity and mortality increase as well as maternal complications such as pre-eclampsia, postpartum haemorrhage and caesarean delivery.^18^–^20^ Women with high body mass index (BMI) and prolonged pregnancy are therefore becoming an increasingly prevalent clinical problem. To reduce the risk of perinatal mortality in prolonged pregnancy, the National Institute for Clinical Excellence antenatal care guidelines recommend that IOL is offered between 41 and 42 weeks of gestation and, if this is declined, twice weekly cardiotocography and ultrasound assessment of liquor volume are recommended after 42 weeks of gestation.^21^ Management of prolonged pregnancies in obese women, however, is difficult because IOL is associated with a high risk of caesarean section and its attendant complications of infection, haemorrhage and thrombosis whereas conservative management is associated with an increased risk of perinatal mortality. The clinician managing an obese woman with a prolonged pregnancy therefore faces the dilemma of whether to; induce her and risk caesarean section delivery and its complications, which can include maternal death, to book an elective caesarean section and thereby reduce the increased risks associated with emergency caesarean section, or to wait so as to maximise the chance of spontaneous labour, thereby reducing the risk of caesarean section but increasing the risk of fetal death, even with outpatient monitoring. There are few published data that inform the clinician and their patients as to the prevalence of complications with each of these options. The aim of this study was therefore to assess the size of the risk of delivery complications following IOL in obese pregnant women with prolonged pregnancies in a large UK maternity unit (Liverpool Women's NHS Foundation Trust).

We initially assessed the risk of prolonged pregnancy with high maternal BMI in our study population. Next, we examined our primary outcome measure of the mode of delivery following IOL for prolonged pregnancy in obese women versus women of normal weight. Then we investigated our secondary outcome measure of the incidence of delivery complications in obese women with prolonged pregnancy following IOL, compared with their normal weight counterparts.

## Methods

We performed a retrospective (historical) cohort study using data gathered from the obstetric records of women with a singleton pregnancy delivering after 24 weeks of gestation between January 2004 and December 2008 at Liverpool Women's Hospital NHS Foundation Trust, Liverpool, UK. This trust provides maternity care for over 8000 deliveries per annum. Anonymous data were retrieved from the Meditech database and the labour and delivery records, which were entered at booking (approximately 12 weeks of gestation) and immediately after delivery. The maternal variables that we assessed were; age, race, height and weight at booking, parity, smoking status, gestation at delivery, delivery outcome including onset of delivery, mode of delivery, reason for delivery mode, labour length (first, second and third stages), estimated blood loss, second and third degree tears and episiotomy. Neonatal characteristics included sex, birthweight, Apgar score at 1 and 5 minutes after delivery, cord blood pH and the incidence of shoulder dystocia and stillbirth. Data were analysed using Statistical Package for the Social Sciences (SPSS), Version 16 (SPSS Inc., Chicago, IL, USA). Ethical approval was sought before commencing the study (Liverpool REC Ref 08/H1005/13).

Maternal BMI was calculated based upon maternal height and weight measurements provided during pregnancy booking between gestational weeks 10 and 12. Women were grouped into the following six BMI categories: ≤19.9 kg/m^2^ (underweight), 20–24.9 kg/m^2^ (normal weight) 25–29.9 kg/m^2^ (overweight), 30–34.9 kg/m^2^ (obese), 35–39.9 kg/m^2^ (very obese) and >40 kg/m^2^ (morbidly obese). In some analyses, to enable stringent exclusion criteria to be applied (and therefore smaller sample sizes) all women with a BMI of ≥30 kg/m^2^ were combined and classified as obese. Gestational age records were based upon an ultrasound scan taken at booking. Prolonged pregnancy was defined as delivery on or after 290 days (41^+3^ weeks) of gestation, as the hospital protocol is to refer women to a dedicated prolonged pregnancy clinic at 290 days. At the clinic, women have a cardiotocogram and ultrasound assessment of liquor volume. If these are normal, then women are given a choice between immediate induction or alternate day fetal surveillance. The majority of women (>90%) choose induction. The hospital protocol for IOL was the same in obese and non-obese women. Term delivery was defined as reaching 37–41^+2^ weeks (259–289 days), while preterm birth was defined as any birth before 37 completed weeks (259 days) of gestation.

The main outcome measures included length of gestation, prolonged pregnancy and delivery outcomes such as need for caesarean section following IOL. Measures used to assess the rate of complications in obese women with prolonged pregnancy undergoing IOL included length of labour, estimated blood loss and incidence of postpartum haemorrhage (defined as an estimated blood loss of >500 ml for vaginal deliveries and >1000 ml for delivery by caesarean section), second- and third-degree tear, episiotomy, retained placenta (defined as third stage >30 minutes), macrosomia (>4.5 kg), cord blood pH <7.0 and <7.2, Apgar score <7 after 5 minutes, incidence of shoulder dystocia and stillbirth.

### Exclusion criteria

**Figure 1:**
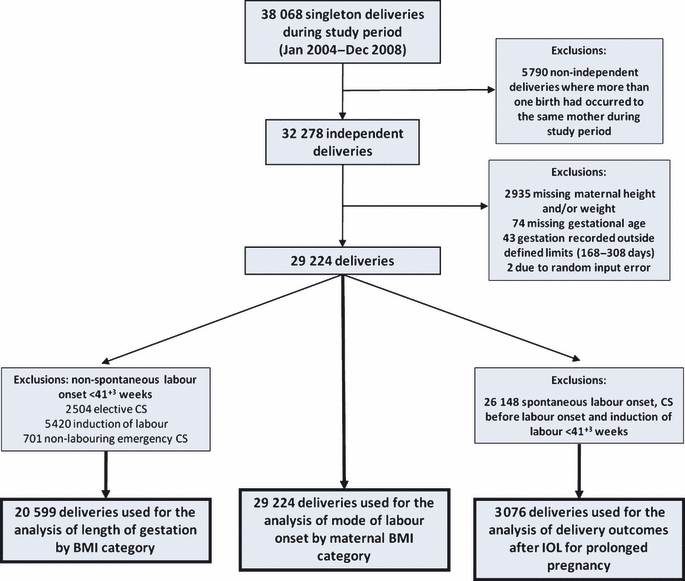
Number of singleton deliveries during the study period and the application of exclusion and inclusion criteria for each study sample.

Strict exclusion criteria were applied for each question addressed (Figure 1). For all investigations we only included each mother once so the 5790 subsequent pregnancies to the same mothers during the study period were excluded from the cohort. Women with missing data for gestation, height or weight and those with gestations <168 days or >308 days or faulty data (random input error) (*n* = 3054) were also excluded.

To examine the risk of prolonged pregnancy in our population of women we also excluded women with nonspontaneous labour onset before 290 days (*n* = 8625) because these pregnancies had an intervention preventing the true gestation from being ascertained.

For analysis of mode of delivery following IOL, and to examine the effect of high BMI on delivery complications following IOL for women with a prolonged pregnancy, only women with a pregnancy reaching 290 days were included in this study cohort because this follows the hospital's protocol for IOL for prolonged pregnancy. Therefore we excluded women who would not present to antenatal clinic with prolonged pregnancy because they would have been delivered electively early.

### Statistical analysis

This is a pragmatic analysis of clinical data so the clinically most useful approach was used and comparisons were made between obese and normal weight women. We examined length of gestation by booking BMI group by Kruskal–Wallis test. Bivariate analyses were performed and chi-square statistic was computed to assess the association between maternal characteristics and delivery for each gestational age group. Multivariable logistic regression was performed to assess the risk of prolonged pregnancy with increased BMI while controlling for confounding variables noted from the bivariate analyses. Term pregnancies and women of normal weight were used as the reference categories in the regression model. Variables included in the model were maternal age, race, parity, maternal hypertension or diabetes (gestational and pregestational) and smoking status. Survival curves were constructed for each BMI category where delivery was the outcome event. Curves were compared by the Log-rank test and a statistical difference was taken at *P*< 0.05 level of significance.

To examine mode of delivery following IOL in women with a prolonged pregnancy and to compare their outcomes with those of their normal weight counterparts, vaginal, assisted vaginal (use of forceps or ventouse) and caesarean section delivery rates after IOL were examined by BMI category and chi-square tests were performed. To examine how the incidence of delivery complications after IOL in obese women with a prolonged pregnancy compared with their normal weight counterparts, Kruskal–Wallis or chi-square tests were performed. Outcomes were assessed according to mode of delivery, i.e. vaginal or caesarean section. For these analyses, all women with a BMI ≥ 30 were classified as obese and only women having IOL ≥ 290 days were included in the analysis. Analyses stratified by parity group (primiparous or multiparous) were also computed provided we retained statistical power. For all statistical analyses *P*< 0.05 was taken as the level of significance.

## Results

### Risk of prolonged pregnancy in our sample population

**Figure 2:**
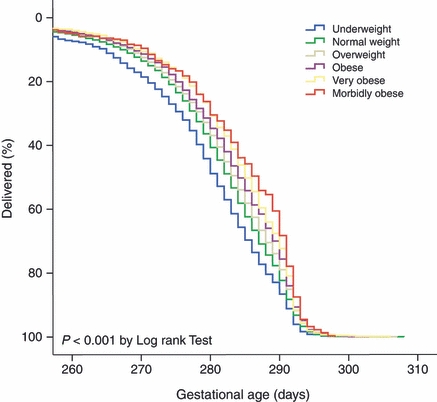
Survival curves for the BMI categories where the event is delivery.

The study cohort used to examine the effect of maternal BMI on gestational length and risk of prolonged pregnancy consisted of 20 599 singleton deliveries resulting in spontaneous labour or having labour induction ≥290 days (see methods and Figure 1 for exclusion criteria). The study demographics are summarised in Table 1. Median length of gestation assessed by Kruskal–Wallis test indicated that a greater maternal BMI at booking was associated with longer gestation (*P*< 0.001). Median (interquartile range) gestation at delivery were as follows: underweight, 281 days (274–287 days); normal weight, 283 days (276–289 days); overweight, 284 days (277–290 days); obese, 284 days (277–290 days); very obese, 286 days (279–291 days) and morbidly obese, 287 days (279–291 days). This difference was reflected in the survival analysis, which identified significantly different survival curves for each of the BMI categories. Women of higher maternal BMI at booking were shifted towards a later delivery (*P*< 0.001, Log-rank test) (Figure 2).

**Table 1:** Maternal characteristics for the study cohort used to investigate length of gestation and risk of prolonged pregnancy by maternal BMI category

Study sample descriptives (*n* = 20 599)	
Age (years) mean (SD)	28.6 (6.1)
Height (m) mean (SD)	1.64 (0.7)
Weight (kg) median (IQR)	65 (58–75)
Gestational age (days) median (IQR)	283 (276–289)
**BMI, *n* (%)**	
Underweight	2145 (10.4)
Normal	9837 (47.8)
Overweight	5470 (26.6)
Obese	2112 (10.3)
Very obese	723 (3.5)
Morbidly obese	312 (1.5)
**Race, *n* (%)**	
White	17 836 (86.6)
Black	658 (3.2)
Asian	592 (2.9)
Other	891 (4.3)
Unknown	622 (3.0)
**Parity, *n* (%)**	
Primiparous	11 530 (56.0)
One previous pregnancy	5627 (27.3)
More than one previous pregnancy	3437 (16.7)
Unknown	5 (0.01)
**Smoking status, *n* (%)**	
Non-smoker	12 540 (60.9)
Stopped since pregnant	2370 (11.5)
Smoker (>1 per day)	5689 (27.6)
**Hypertension, *n* (%)**	642 (3.1)
**Diabetes mellitus, *n* (%)**	45 (0.2)

Prolongation of pregnancy was seen in 30.0% of obese women compared with 22.3% or 17.1% of normal weight and underweight women, respectively. Moreover, very obese or morbidly obese women had further increased incidence of prolonged pregnancy (32.4 and 39.4%, respectively). Higher maternal age, white race and lower parity were also shown to be associated with prolonged pregnancy. Smoking during pregnancy resulted in a reduced incidence of prolonged pregnancy and greatly increased the number of women delivering preterm. Preterm women also tended to have a higher incidence of hypertension and diabetes (gestational or pregestational).

To account for potential confounding factors, we next performed multivariate logistic regression analysis to examine the likelihood of prolonged pregnancy with increased maternal BMI at booking (Table 2). Compared with women in the normal BMI group and using term deliveries as a reference category, women in the higher BMI categories had significantly increased odds of having a prolonged pregnancy despite adjusting for the influences of maternal age, race, parity, maternal hypertension, diabetes and smoking status. Conversely, women in the underweight category appeared to be protected from prolonged pregnancy, having significantly decreased odds in comparison to their normal weight counterparts but they were at increased risk of delivering preterm (Table 2). Having established the effects of BMI on gestational length in our cohort, we went on to assess delivery outcomes in obese women following IOL for prolonged pregnancy.

**Table 2:** Adjusted odds ratios and 95% confidence intervals calculated for prolonged pregnancy or preterm delivery according to maternal BMI category at pregnancy booking in comparison to a normal BMI of 20–24.9 kg/m^2^

	Maternal BMI group at pregnancy booking					
	
	Underweight (*n* = 2087)	Normal weight (*n* = 9530)	Overweight (*n* = 5294)	Obese (*n* = 2051)	Very obese (*n* = 707)	Morbidly obese (*n* = 303)
Preterm(*n* = 907)	1.33 (1.09–1.62)*	1.00	0.81 (0.68–0.97)*	0.92 (0.72–1.17)	0.84 (0.56–1.28)	1.25 (0.72—0.19)
Term(*n* = 14 229)	Ref	1.00	Ref	Ref	Ref	Ref
Prolonged(*n* = 4836)	0.75 (0.66–0.85)*	1.00	1.24 (1.14–1.34)*	1.52 (1.37–1.70)*	1.75 (1.48–2.07)*	2.27 (1.78–2.89)*

Values are adjusted odds ratio (AOR) with 95% CI in parentheses. Analyses controlled for the following variables: maternal age, maternal race, parity, hypertension (pregestational or gestational), diabetes mellitus (pregestational or gestational) and smoking status. Normal weight and term pregnancy were used as the reference categories.

* An AOR >1 indicates a significantly increased risk of prolonged pregnancy or preterm delivery whereas an AOR <1 indicates significantly less risk compared with women of normal weight (*P* < 0.05).

### Primary outcome measure: mode of delivery following induction of labour

The overall incidence of labour induction by maternal BMI category (all gestations) was investigated. Data from 29 224 women were eligible for inclusion (see Figure 1). A total of 8497 women had their labours induced (29.1%). As maternal BMI increased, there was a dose-dependent increase in number of women having IOL (Table 3) such that 34.4% of obese women had IOL, compared with 30.5 and 26.2% of overweight and normal weight women, respectively, demonstrating that obese women more often required labour induction than their respective counterparts of normal weight.

**Table 3:** Mode of labour onset for deliveries at all gestations tabulated according to maternal BMI category at booking (*n* = 29 224)

BMI group	Mode of labour onset			
	
	Spontaneous (*n* = 17 417)	Elective caesarean section (*n* = 2568)	Emergency caesarean section (*n* = 742)	Induction (*n* = 8497)
Underweight (%) (*n* = 2831)	69.0	4.7	2.1	24.2
Normal (%) (*n* = 13 231)	64.1	7.4	2.4	26.2
Overweight (%) (*n* = 7989)	56.9	10.1	2.5	30.5
Obese (%) (*n* = 3303)	50.5	11.7	3.4	34.4
Very obese (%) (*n* = 1267)	43.7	13.3	3.0	40.0
Morbidly obese (%) (*n* = 603)	35.5	16.7	4.1	43.6
Overall (%) (*n* = 29 224)	59.6	8.8	2.5	29.1

The study sample used to address the more specific question; how does mode of delivery following IOL for prolonged pregnancy compare in obese versus normal weight women, comprised 3076 women, all of whom were identified as having IOL at ≥290 days. The overall rate of vaginal delivery after induction was found to be 76.4% (*n* = 2351). Analysis of vaginal delivery or assisted vaginal delivery after induction by BMI group indicated a decrease in vaginal delivery after induction with increasing maternal BMI (Table 4) showing that a greater number of obese women had an induction ending in caesarean section compared with normal weight women who were induced (*P*< 0.001). Similar trends were observed when the study sample was stratified by parity (primiparous versus multiparous women) but rates of vaginal delivery after induction for primiparous women (Table 4) were almost half those of multiparous women (Table 4) at each respective BMI group.

**Table 4:** Mode of delivery outcome following IOL in prolonged pregnancy for women of mixed parity, primiparous women and multiparous women, according to maternal BMI group at pregnancy booking

	Unassisted vaginal delivery	Assisted vaginal delivery	Caesarean section delivery
Delivery mode after IOL all women (*n* = 3076)	(*n* = 1742)	(*n* = 609)	(*n* = 725)

Underweight, (%) (*n* = 189)	61.9	21.7	16.4
Normal weight, (%) (*n* = 1315)	57.9	23.1	18.9
Overweight, (%) (*n* = 887)	54.8	17.0	28.2
Obese, (%) (*n* = 685)	55.0	16.5	28.5
*P* value			<0.001

Delivery mode after IOL for primiparous women (*n* = 2066)	(*n* = 902)	(*n* = 545)	(*n* = 619)

Underweight, (%) (*n* = 149)	54.4	26.8	18.8
Normal weight, (%) (*n* = 910)	46.5	29.7	23.8
Overweight, (%) (*n* = 565)	40.2	23.5	36.3
Obese, (%) (*n* = 442)	38.5	22.9	38.7
*P* value			<0.001

Delivery mode after IOL for multiparous women (*n* = 1010)	(*n* = 840)	(*n* = 64)	(*n* = 106)

Underweight, (%) (*n* = 40)	90.0	2.5	7.5
Normal weight, (%) (*n* = 405)	83.9	8.2	7.9
Overweight, (%) (*n* = 322)	80.4	5.6	14.0
Obese, (%) (*n* = 243)	85.2	4.9	9.9
*P* value			0.998

Assisted vaginal delivery is defined as vaginal delivery requiring forceps or ventouse.

*P* < 0.05 indicates that the trend was significant by chi-square test.

Analysis of the reason for delivery by caesarean section following induction highlighted that women who were obese had a greater incidence of ‘unsuccessful induction’ noted as reason for caesarean section compared with their normal weight counterparts (8.8% obese versus 3.6% normal weight; Table 5). The hospital definition of unsuccessful induction is no dilation of the cervix after 12 hours of vaginal prostaglandin and 10 hours of intravenous syntocinon. Caesarean section for reasons such as failure to progress and fetal distress following induction remained fairly constant between BMI groups (Table 5).

**Table 5:** Reason for caesarean section following IOL for all women with prolonged pregnancy according to maternal BMI category at pregnancy booking (*n* = 725)

	Maternal BMI category			
	
	Underweight (*n* = 31)	Normal weight (*n* = 249)	Overweight (*n* = 250)	Obese (*n* = 195)
Cord presentation (%)	3.2	1.6	0.0	1.6
Cephalopelvic disproportion (%)	3.2	2.0	0.8	0.5
Deep transverse arrest (%)	3.2	2.0	2.0	1.5
Delay stage 1 (%)	35.5	36.5	36.0	36.4
Delay stage 2 (%)	9.7	10.8	8.8	10.3
Fetal distress (%)	38.7	35.3	41.6	39.0
Unsuccessful induction (%)	3.2	3.6	8.0	8.7
Unsuccessful forceps/ventouse (%)	0.0	2.8	1.6	1.5
Maternal request (%)	3.2	1.6	0.8	0.5
Other* (%)	3.2	5.2	0.4	2.6

* Other includes: unstable position (2.0%), undiagnosed breech (0.5%) previous difficult vaginal delivery (0.5%), cervical stenosis (0.4%), antepartum haemorrhage (0.4%), previous caesarean section (0.4%), and placenta praevia (0.8%).

### Secondary outcome measures

For vaginal deliveries (unassisted and assisted combined) after IOL, no difference in median length of first stage of labour with higher BMI was reported and obese women appeared to have a significantly shorter median second stage of labour compared with normal weight women (*P* ≤ 0.001 Table 6). Bivariate analyses identified a lower rate of episiotomy for obese women but a higher rate of second-degree tearing. However, we found no significant difference in incidence of third-degree tearing with higher BMI (Table 6). Incidence of retained placenta was observed at a lower rate for obese women compared with normal weight women. Postpartum haemorrhage in vaginal deliveries, defined as estimated blood loss >500 ml, was no different between BMI groups. There was a nonsignificant trend for estimated blood loss >1000 ml in obese women compared with women of normal weight. This trend towards increased incidence of postpartum haemorrhage was also observed when examining delivery by caesarean section but again was not significant (Table 6).

**Table 6:** Labour and delivery outcomes by vaginal or caesarean section delivery and neonatal outcomes for all prolonged pregnancies (mixed parity) following IOL by maternal BMI category

	Underweight	Normal	Overweight	Obese	*P* value
**Pregnancy outcome by maternal BMI group (vaginal deliveries only *n* = 2351)**					
Length stage 1 (hours) Median (IQR)	4.8 (3.1–7.1)	4.3 (2.6–6.9)	4.1 (2.6–6.8)	4.3 (2.4–7.2)	0.424
Length stage 2 (hours) Median (IQR)	1.0 (0.5–1.9)	0.9 (0.3–2.1)	0.6 (0.3–1.6)	0.5 (0.2–1.6)	<0.001*
Retained placenta, *n*, (%)	0 (0.0)	44 (4.1)	22 (3.5)	13 (2.7)	0.042*
Second-degree tear, *n* (%)	32 (20.3)	213 (20.0)	130 (20.4)	135 (27.6)	0.006*
Third-degree tear, *n* (%)	5 (3.2)	40 (3.8)	22 (3.5)	10 (2.0)	0.363
Episiotomy, *n* (%)	42 (26.6)	310 (29.1)	152 (23.9)	105 (21.4)	0.007*
PPH (>500 ml), *n* (%)	23 (14.6)	182 (17.1)	102 (16.0)	74 (15.1)	0.714
PPH (>1000 ml), *n* (%)	5 (3.2)	32 (3.0)	13 (2.0)	18 (3.7)	0.424
**Pregnancy outcome by maternal BMI group (caesarean section deliveries only, *n* = 725)**					
PPH (>500 ml), *n* (%)	25 (80.6)	192 (77.1)	199 (79.6)	165 (84.6)	0.268
PPH (>1000 ml), *n* (%)	1 (3.2)	20 (8.0)	25 (10.0)	20 (10.3)	0.533
**Neonatal outcome by maternal BMI group**					
Macrosomia, *n* (%) (*n* = 2995)	7 (3.8)	55 (4.3)	46 (5.3)	53 (8.0)	0.005*
Cord blood pH <7.2, *n* (%) (*n* = 2688)	29 (17.9)	276 (24.2)	201 (25.8)	141 (23.4)	0.183
Cord blood pH <7.0, *n* (%) (*n* = 2688)	2 (1.2)	6 (0.5)	9 (1.2)	7 (1.2)	0.382
Apgar <7 (5-minutes), *n* (%) (*n* = 3067)	5 (2.6)	16 (1.2)	21 (2.4)	8 (1.2)	0.090
Shoulder dystocia, *n* (%) (*n* = 3002)	3 (1.6)	32 (2.5)	24 (2.8)	18 (2.7)	0.827
Stillbirth, *n* (*n* = 3076)	0	0	1	1	0.538**

PPH, postpartum haemorrhage.

Values are presented as medians and interquartile range (IQR) for lengths of labour or total counts (*n*) and percentages (%) for all other analyses. Values presented in bold with * indicate that the trend was significant (*P*< 0.05 by Kruskal–Wallis test or chi-square test).

** Indicates loss of power.

In subgroup analysis limited to primiparous women (Table 7), similar trends to the mixed parity group were identified but the significantly shorter second stage of labour and reduced rates of episiotomy for obese women were lost. Again, there was a nonsignificant trend for increased postpartum blood loss in the obese primiparous group (Table 7) so both primiparous and mixed parity obese women were at no greater risk for postpartum haemorrhage than women of normal weight (Table 7). For analysis of outcomes for multiparous women following IOL (Table 8), no significant trends in length of first stage of labour, incidence of retained placenta or incidence of second- or third-degree tearing or postpartum blood loss were observed with increasing maternal BMI. However, incidence of episiotomy was reduced and length of second stage of labour was shorter as maternal BMI increased (Table 8).

**Table 7:** Labour and delivery outcomes by vaginal or caesarean section delivery and neonatal outcomes for all primiparous women with a prolonged pregnancy following IOL by maternal BMI category

	Underweight	Normal	Overweight	Obese	*P* value
**Pregnancy outcome by maternal BMI group (vaginal deliveries only *n* = 1447)**					
Length stage 1 (hours) Median (IQR)	5.3 (3.7–7.5)	5.3 (3.5–8.0)	5.5 (3.8–8.0)	5.8 (3.5–8.5)	0.630
Length stage 2 (hours) Median (IQR)	1.2 (0.6–2.0)	1.4 (0.7–2.6)	1.3 (0.6–2.3)	1.3 (0.5–2.5)	0.158
Retained placenta, *n* (%)	0 (0.0)	25 (3.6)	11 (3.1)	6 (2.2)	0.152
Second-degree tear, *n* (%)	26 (21.5)	134 (19.3)	86 (23.9)	87 (32.1)	0.001*
Third-degree tear, *n* (%)	4 (3.3)	37 (5.3)	17 (4.7)	7 (2.6)	0.277
Episiotomy, *n* (%)	42 (34.7)	271 (39.0)	128 (35.6)	93 (34.3)	0.455
PPH (>500 ml), *n* (%)	22 (18.2)	157 (22.6)	78 (21.7)	63 (23.2)	0.701
PPH (>1000 ml), *n* (%)	5 (4.1)	24 (3.5)	10 (2.8)	17 (6.3)	0.127
**Pregnancy outcome by maternal BMI group (caesarean section deliveries only, *n* = 619)**					
PPH (>500 ml), *n* (%)	22 (78.6)	169 (77.9)	164 (80.0)	145 (84.8)	0.383
PPH (>1000 ml), *n* (%)	1 (3.6)	17 (7.8)	20 (9.8)	15 (8.8)	0.700
**Neonatal outcome by maternal BMI group**					
Macrosomia, *n* (%) (*n* = 2016)	3 (2.1)	31 (3.5)	22 (4.0)	27 (6.3)	0.052
Cord blood pH <7.2, *n* (%) (*n* = 1810)	28 (21.7)	211 (26.8)	161 (32.2)	99 (25.2)	0.031*
Cord blood pH <7.0, *n* (%) (*n*= 1810)	2 (1.6)	4 (0.5)	7 (1.4)	5 (1.3)	0.328**
Apgar <7 (5-minutes), *n* (%) (*n* = 2063)	5 (3.4)	14 (1.5)	17 (3.0)	6 (1.4)	0.105
Shoulder dystocia, *n* (%) (*n* = 1999)	2 (1.4)	18 (2.0)	11 (2.0)	11 (2.6)	0.827
Stillbirth, *n* (*n* = 2066)	0	0	1	1	0.538**

PPH, postpartum haemorrhage.

Values are presented as medians and interquartile range (IQR) for lengths of labour or total counts (*n*) and percentages (%) for all other analyses. Values presented in bold with * indicate that the trend was significant (*P*< 0.05 by Kruskal–Wallis test or chi-square test).

** Indicates loss of power.

**Table 8:** Labour and delivery outcomes by vaginal or caesarean section delivery and neonatal outcomes for all multiparous women with a prolonged pregnancy following IOL by maternal BMI category

	Underweight	Normal	Overweight	Obese	*P* value
**Pregnancy outcome by maternal BMI group (vaginal deliveries only *n*= 904)**					
Length stage 1 (hours) Median (IQR)	2.9 (1.4–5.0)	2.9 (1.7–4.5)	3.0 (1.7–4.4)	2.9 (1.6–4.7)	0.998
Length stage 2 (hours) Median (IQR)	0.4 (0.1–0.8)	0.3 (0.2–0.7)	0.3 (0.2–0.5)	0.3 (0.1–0.4)	<0.001*
Retained placenta, *n* (%)	0 (0.0)	19 (5.1)	11 (4.0)	7 (3.2)	0.386
Second-degree tear, *n* (%)	6 (16.2)	79 (21.3)	44 (15.9)	48 (21.9)	0.249
Third-degree tear, *n* (%)	1 (2.7)	3 (0.8)	5 (1.8)	3 (1.4)	0.619**
Episiotomy, *n* (%)	0 (0.0)	39 (10.5)	24 (8.7)	12 (5.5)	0.044*
PPH (>500 ml), *n* (%)	1 (2.7)	25 (6.7)	24 (8.7)	11 (5.0)	0.306
PPH (>1000 ml), *n* (%)	0 (0.0)	8 (2.2)	3 (1.1)	1 (0.5)	0.279**
**Pregnancy outcome by maternal BMI group (caesarean section deliveries only *n*= 106)**					
PPH (>500 ml), *n* (%)	3 (100.0)	23 (71.9)	35 (77.8)	20 (83.3)	0.586**
PPH (>1000 ml), *n* (%)	0 (0.0)	3 (23.1)	5 (11.1)	5 (20.8)	0.509**
**Neonatal outcome by maternal BMI group**					
Macrosomia, *n* (%) (*n* = 979)	4 (10.0)	24 (6.1)	24 (7.7)	26 (11.1)	0.159
Cord blood pH <7.2, *n* (%) (*n* = 878)	1 (3.0)	65 (18.3)	40 (14.4)	42 (20.0)	0.053
Cord blood pH <7.0, *n* (%) (*n* = 878)	0 (0.0)	2 (0.6)	2 (0.7)	2 (0.9)	0.913**
Apgar <7 (5-minutes), *n* (%) (*n* = 1004)	0 (0.0)	2 (0.5)	4 (1.2)	2 (0.8)	0.663**
Shoulder dystocia, *n* (%) (*n* = 1010)	1 (2.5)	14 (3.5)	13 (4.0)	7 (2.9)	0.877**
Stillbirth, *n* (*n* = 1010)	0	0	0	0	—

PPH, postpartum haemorrhage.

Values are presented as medians and interquartile range (IQR) for lengths of labour or total counts (*n*) and percentages (%) for all other analyses. Values presented in bold with * indicate that the trend was significant (*P*< 0.05 by Kruskal–Wallis test or chi-square test).

** Indicates loss of power.

— Indicates that all values were zero so no chi-square statistic was computed.

Analysis of neonatal outcomes from all deliveries after IOL for prolonged pregnancy found a dose dependent percentage increase in fetal macrosomia with increasing BMI category (*P*= 0.005, Table 6). This trend was also true for primiparous women but just failed to reach significance (*P*= 0.052, Table 7) and no trend was observed for analysis restricted to multiparous women (Table 8). There was no difference in incidence of cord blood pH <7.2, <7.0 or apgar scores <7 after 5 minutes for the mixed parity group (Table 6) or multiparous group (Table 8), however neonates from underweight, primiparous mothers had an increased incidence of cord blood pH <7.2 compared with women of normal BMI or greater (Table 7). Statistical power for the analysis of cord blood pH <7.0 was lost when outcomes were stratified according to parity. There was also a trend towards increased incidence of shoulder dystocia with increasing BMI for primiparous women (Table 7); however, this was not shown to be significant and no trend was observed for multiparous women (Table 8). Overall, incidence of stillbirth was low (two stillbirths) and although these occurred to one overweight and one obese primiparous woman, there was insufficient statistical power to assess the significance between the different BMI groups.

## Discussion

The current obesity epidemic presents frequent challenges to the obstetrician. Our study is consistent with those of others who found that maternal obesity is a significant risk factor for post-term delivery.^8^,^10^,^11^,^13^,^14^,^17^ In this study we extend previous findings by assessing the effect of high maternal BMI on labour and delivery complications following IOL in a large group of women with prolonged pregnancies booked to deliver at a tertiary referral hospital.

We demonstrate that more obese women required IOL and that IOL for these women was associated with increased rates of caesarean section delivery. This relationship also held true when specifically examining the outcomes of women with prolonged pregnancy (overall rates of induction needing caesarean section for was 28.4% for obese women compared with 18.9% for normal weight women). Although this 28.4% caesarean section rate is high, it is also important to realise that over 70% of obese pregnant women with prolonged pregnancy delivered vaginally. Hence IOL is a reasonable way to avoid caesarean section and elective caesarean section is not indicated in all women. Similarly, while vaginal delivery in obese pregnant women is potentially hazardous, our data on vaginal delivery were reassuring. A median length of first stage of labour of 4 hours and a second stage of labour of 1 hour, third-degree tear rate of 2%, and significant postpartum haemorrhage rate of 4% are probably all acceptable to women and their clinicians. These data are important to the clinician in the antenatal clinic when faced with an obese woman with prolonged pregnancy. If induced, she has a 60% chance of achieving a vaginal delivery if primiparous and a 90% chance if multiparous, without an increase in other complications of labour with modern intrapartum care. Importantly, during the study period the majority of out-of-hours cover was by junior staff. The small number of complications following induction in obese women therefore suggests that junior staff with on-call consultant cover can provide a good service to these women.

The association between increasing maternal BMI and macrosomia confirms previous reports.^6^,^8^,^13^,^16^,^17^,^22^ However, despite increasing macrosomia with increasing BMI there was no increase in cord blood pH <7.2 or <7.0 or low Apgar score or incidence of shoulder dystocia with increasing BMI for women of mixed parity. This finding is again reassuring, reflects good obstetric care and reaffirms the place of IOL in the management of obese pregnant women with prolonged pregnancy.

Some reports,^22^–^24^ but not all,^13^,^16^ have associated obesity with a risk for preterm delivery. Our data suggest that it is underweight women who are more likely to deliver preterm. Although obesity is associated with pre-eclampsia and elective preterm delivery,^24^ obesity is not associated with spontaneous preterm delivery. In an attempt to examine this further we compared gestational age outcome by BMI with the inclusion of women undergoing labour induction before 290 days. In this analysis (not shown), the different survival curves for each BMI category persisted but were shifted to the left because of an increased incidence of preterm delivery among underweight women. The shift in the survival curves also reflects the fact that numerically, more obese women have prolonged pregnancies than have preterm deliveries.

The association of obesity with prolonged pregnancy, but not preterm labour, implies that obesity is associated with uterine quiescence or a suppression of myometrial activity. This study adds to our previously reported findings of an association between obesity and reduced myometrial contractility by both clinical and laboratory markers.^7^ However, the lack of association between increasing BMI and length of first and second stages of labour, higher caesarean section for failure to progress and higher incidence of retained placenta mitigate against an underlying aetiology of poor myometrial contractility in obese women with prolonged pregnancy in established labour. We suggest that the effect of obesity on myometrial activity is more important in delaying the onset of labour than in reducing myometrial function once labour is established.

### Study limitations

Some limitations of this study should be noted. This is a pragmatic analysis of a clinical data set and informs the clinician in the antenatal clinic as to the risks of managing prolonged pregnancies in obese women. This means it is affected by clinical decision-making and there will inevitably be some caveats, e.g. women judged to be at high risk for stillbirth or macrosomia may have been electively delivered before 40 weeks gestation and therefore excluded from the analysis. Similarly, cervical assessment may have influenced the decision not to induce some women. The cohort analyses of prolonged pregnancy may therefore reflect those women who may have been particularly difficult to induce. As a retrospective and observational study it is based upon data collected from within a pre-existing database and while women with missing data for height, weight and gestational age were excluded from our analyses, there were some women with other variables missing from the data set. We were unable to assess the effect of maternal BMI on incidence of stillbirth because the number was small. Information on some important confounding factors, for example, social class, medical history and paternal race, were also omitted from the data set, which may have influenced the relationships between obesity and prolonged pregnancy outcomes.^14^

Our study used maternal booking BMI data because it is routinely available and well understood; other methods may more accurately determine maternal adiposity, and take account of maternal height (an independent risk factor for caesarean section^25^) but these are not as readily available. It would also have been of interest to have data on weight gain in pregnancy for our cohort because this has also been shown to be associated with longer gestation and increased risk of post-term delivery;^8^,^10^,^11^ however, women are not routinely weighed after booking in current UK practise.

Retrospective studies examining mode of delivery following IOL have also been criticised for their nonrandomised design approach and instead, prospective randomised trials of IOL for prolonged pregnancies versus other gestations are argued to be more appropriate. In their 2006 study, Caughey et al.^26^ found a decrease in caesarean section delivery after induction, owing to the effect of expectant management of prolonged pregnancy. We examined outcomes by IOL at different durations of gestation versus expectant management according to maternal BMI category, but we did not have sufficient statistical power to assess this association and so the data are not shown. Outcomes for obese women following IOL at gestations other than 290 days warrants further examination. We selected our time point because this is the gestation at which women under our care are referred to a designated prolonged pregnancy clinic. At the clinic, a computer-analysed cardiotocogram is performed and a measurement of amniotic fluid index is obtained. If either of these is abnormal then immediate induction is advised. If they are normal then women are offered a choice between IOL or continued surveillance. Most women choose induction because of maternal discomfort associated with prolonged pregnancy. Hence there were insufficient spontaneous labours after 41^+3^ weeks to allow meaningful analysis. It remains to be seen whether an earlier IOL policy for obese women would be an effective strategy to prevent stillbirth or the exact increase in caesarean section rate that would follow IOL.

In this study we confirm that maternal obesity is associated with an increased risk of prolonged pregnancy, but not preterm delivery, and suggest that delayed onset of labour is the underlying reason. With the increasing national rate of maternal obesity, if a causal relationship is true, then we would expect an increase in the prevalence of deliveries beyond term, and specifically an increase in the number of obese women presenting with a prolonged pregnancy. One of the common dilemmas in the management of the obese pregnant woman with prolonged pregnancy is how to balance the maternal risks of IOL, including caesarean section and postpartum haemorrhage, with the fetal risks of perinatal morbidity associated with prolonged gestation. For this reason, the clinical ramifications of this study are important. We have examined delivery outcomes after labour induction in obese pregnant women and more specifically the effect of higher maternal BMI on delivery outcome following induction for prolonged pregnancy in a large NHS hospital where antenatal care is aimed at achieving vaginal delivery. In conclusion, our analysis of deliveries resulting from IOL in prolonged pregnancies found an increased rate of caesarean section in obese women compared with women of normal weight. However, the majority of prolonged pregnancies in obese women, including those of primiparous women, were delivered vaginally with normal lengths of labour and acceptable rates of labour complications. We suggest that IOL in obese women with prolonged pregnancy is a reasonable and safe management option that can be embarked upon with clinical confidence and optimistic counselling of women.

## References

[1] World Health Organisation Fact sheet: obesity and overweight. http://www.who.int/mediacentre/factsheets/fs311/en/index.html.

[2] Ramsay JE, Greer I, Sattar N (2006). ABC of obesity. Obesity and reproduction. BMJ.

[3] Heslehurst N, Simpson H, Ells LJ, Rankin J, Wilkinson J, Lang R (2008). The impact of maternal BMI status on pregnancy outcomes with immediate short-term obstetric resource implications: a meta-analysis. Obes Rev.

[4] Heslehurst N, Rankin J, Wilkinson JR, Summerbell CD (2010). A nationally representative study of maternal obesity in England, UK: trends in incidence and demographic inequalities in 619 323 births, 1989–2007. Int J Obes (Lond).

[5] Kanagalingam MG, Forouhi NG, Greer IA, Sattar N (2005). Changes in booking body mass index over a decade: retrospective analysis from a Glasgow Maternity Hospital. BJOG.

[6] Cedergren MI (2004). Maternal morbid obesity and the risk of adverse pregnancy outcome. Obstet Gynecol.

[7] Zhang J, Bricker L, Wray S, Quenby S (2007). Poor uterine contractility in obese women. BJOG.

[8] Johnson JW, Longmate JA, Frentzen B (1992). Excessive maternal weight and pregnancy outcome. Am J Obstet Gynecol.

[9] Nohr EA, Bech BH, Davies MJ, Frydenberg M, Henriksen TB, Olsen J (2005). Prepregnancy obesity and fetal death: a study within the Danish National Birth Cohort. Obstet Gynecol.

[10] Denison FC, Price J, Graham C, Wild S, Liston WA (2008). Maternal obesity, length of gestation, risk of postdates pregnancy and spontaneous onset of labour at term. BJOG.

[11] Stotland NE, Washington AE, Caughey AB (2007). Prepregnancy body mass index and the length of gestation at term. Am J Obstet Gynecol.

[12] Vahratian A, Zhang J, Troendle JF, Savitz DA, Siega-Riz AM (2004). Maternal prepregnancy overweight and obesity and the pattern of labor progression in term nulliparous women. Obstet Gynecol.

[13] Khashan AS, Kenny LC (2009). The effects of maternal body mass index on pregnancy outcome. Eur J Epidemiol.

[14] Caughey AB, Stotland NE, Washington AE, Escobar GJ (2009). Who is at risk for prolonged and postterm pregnancy?. Am J Obstet Gynecol.

[15] Graves BW, DeJoy SA, Heath A, Pekow P (2006). Maternal body mass index, delivery route, and induction of labor in a midwifery caseload. J Midwifery Womens Health.

[16] Sebire NJ, Jolly M, Harris JP, Wadsworth J, Joffe M, Beard RW (2001). Maternal obesity and pregnancy outcome: a study of 287,213 pregnancies in London. Int J Obes Relat Metab Disord.

[17] Usha Kiran TS, Hemmadi S, Bethel J, Evans J (2005). Outcome of pregnancy in a woman with an increased body mass index. BJOG.

[18] Olesen AW, Westergaard JG, Olsen J (2003). Perinatal and maternal complications related to postterm delivery: a national register-based study, 1978–1993. Am J Obstet Gynecol.

[19] Caughey AB, Stotland NE, Washington AE, Escobar GJ (2007). Maternal and obstetric complications of pregnancy are associated with increasing gestational age at term. Am J Obstet Gynecol.

[20] Caughey AB, Snegovskikh VV, Norwitz ER (2008). Postterm pregnancy: how can we improve outcomes?. Obstet Gynecol Surv.

[21] NICE antenatal care clinical guideline March 2008. http://guidance.nice.org.uk/CG62.

[22] Driul L, Cacciaguerra G, Citossi A, Martina MD, Peressini L, Marchesoni D (2008). Prepregnancy body mass index and adverse pregnancy outcomes. Arch Gynecol Obstet.

[23] Bhattacharya S, Campbell DM, Liston WA (2007). Effect of Body Mass Index on pregnancy outcomes in nulliparous women delivering singleton babies. BMC Public Health.

[24] Smith GC, Shah I, Pell JP, Crossley JA, Dobbie R (2007). Maternal obesity in early pregnancy and risk of spontaneous and elective preterm deliveries: a retrospective cohort study. Am J Public Health.

[25] Mahmood TA, Campbell DM, Wilson AW (1988). Maternal height, shoe size, and outcome of labour in white primigravidas: a prospective anthropometric study. BMJ.

[26] Caughey AB, Nicholson JM, Cheng YW, Lyell DJ, Washington AE (2006). Induction of labor and cesarean delivery by gestational age. Am J Obstet Gynecol.

